# Short- and long-term direct and indirect costs of illness after ostomy creation – a Swedish nationwide registry study

**DOI:** 10.1186/s12913-023-09850-5

**Published:** 2023-08-08

**Authors:** Eva Carlsson, Annabelle Forsmark, Catarina Sternhufvud, Gina Scheffel, Frederikke B. Andersen, Eva I Persson

**Affiliations:** 1 Department of Surgery, Sahlgrenska University Hospital/Östra, Gothenburg, Sweden; 2 Institute of Health and Care Sciences, Sahlgrenska Academy, University of Gothenburg, Gothenburg, Sweden; 3 PharmaLex Sweden, Gothenburg, Sweden; 4 Coloplast AB, Kungsbacka, Sweden; 5 Coloplast Denmark A/S, Humlebaek, Denmark; 6 Department of Health Sciences, Faculty of Medicine, Lund University, Lund, Sweden

**Keywords:** Colostomy, Cost of illness, Healthcare costs, Ileostomy, Ostomy, Stoma, Urostomy

## Abstract

**Background:**

Despite advance in care of people with an ostomy, related complications remain prevalent. The objective of this study was to examine short- and long-term healthcare resource utilization and associated costs after ostomy creation.

**Methods:**

This observational study was based on retrospectively collected data from national and regional Swedish registries. The population consisted of people living in Sweden, who had an ostomy created. The earliest index date was 1 January 2006, and people were followed for ten years, until death, reversal of temporary ostomy, termination of purchases of ostomy products, or end of study, which was 31 December 2019. Each person with an ostomy was matched with two controls from the general population based on age, gender, and region.

**Results:**

In total, 40,988 persons were included: 19,645 with colostomy, 16,408 with ileostomy, and 4,935 with urostomy. The underlying diseases for colostomy and ileostomy creations were primarily bowel cancer, 50.0% and 55.8% respectively, and additionally inflammatory bowel disease for 20.6% of ileostomies. The underlying cause for urostomy creation was mainly bladder cancer (85.0%). In the first year after ostomy creation (excl. index admission), the total mean healthcare cost was 329,200 SEK per person with colostomy, 330,800 SEK for ileostomy, and 254,100 SEK for urostomy (100 SEK was equivalent to 9.58 EUR). Although the annual mean healthcare cost decreased over time, it remained significantly elevated compared to controls, even after 10 years, with hospitalization being the main cost driver. The artificial opening was responsible for 19.3–22.8% of 30-day readmissions after ostomy creation and for 19.7–21.4% of hospitalizations during the entire study period. For the ileostomy group, dehydration was responsible for 13.0% of 30-day readmissions and 4.5% of hospitalization during the study period.

**Conclusions:**

This study reported a high disease burden for persons with an ostomy. This had a substantial impact on the healthcare cost for at least ten years after ostomy creation. Working ability seemed to be negatively impacted, indicated by increased cost of sickness absence and early retirement. This calls for improved management and support of ostomy care for the benefit of the affected persons and for the cost of society.

**Supplementary Information:**

The online version contains supplementary material available at 10.1186/s12913-023-09850-5.

## Introduction

Severe medical conditions such as bowel, bladder, or gynecological cancers; inflammatory bowel disease (IBD); and diverticulitis may require the creation of an ostomy [1]. An ostomy is an artificial opening on the abdomen connected to the gastrointestinal tract (colostomy or ileostomy) or in the urogenital tract (urostomy), created to allow stool or urine to be removed from the body. It can be created either as a temporary or a permanent solution.

From a total of 10.3 million inhabitants in Sweden [2] it is estimated that about 43,000 people, or 0.4%, are living with an ostomy [3]. This may be expected to increase as a result of population aging. The cornerstone of Swedish ostomy care is that care is performed by specialized stoma care nurses who provide counselling for optimal management of a life with an ostomy [4]. The Swedish healthcare system is largely tax-funded, but healthcare visits are co-paid by the visiting person up to 1,200 SEK during a period of 12 months.

Despite advancements in ostomy creation and care of people with an ostomy, related complications remain prevalent. The most common complications are peristomal skin complications (PSCs), which primarily occur secondary to output leaking under the baseplate of the ostomy appliance and making contact with the skin [5–8]. Other typical complications are parastomal hernia, stomal prolapse and stenosis, dehydration, and bowel obstruction [5–8]. Although the risk of developing complications remains lifelong, the incidence is highest close to discharge with 70–79% of persons with an ileostomy and 53% with a colostomy experiencing complications within two weeks of discharge [9]. Similarly, 30-day readmission rates for people who had an ileostomy created were 20–30% [10], and 15% for persons who had a colostomy created [11].

The management of many ostomy-related complications requires outpatient visits and for severe complications like dehydration and ileus, hospital admission and even acute surgery may be needed. Thus, treatment of ostomy-related complications has a significant impact on the healthcare sector [1, 7, 12–15]. However, the burden of illness for persons with an ostomy created is poorly understood and few studies exist [16–18].

With this background in mind, the objective of this study was to examine the short- and long-term healthcare resource utilization and the direct and indirect costs after ostomy creation in Sweden compared to matched controls from the general population.

## Methods

This study was a descriptive, observational, nationwide study based on retrospectively collected data from Swedish national and regional registries, which focused on the healthcare resource utilization and costs up to 10 years after the creation of an ostomy.

### Registry data sources

Data were extracted by linking the relevant registries based on the unique personal identification number assigned to each Swedish resident at birth or immigration to Sweden. An overview of the data sources is outlined in Table S1. The National Patient Register informed data on demographics, clinical outcomes, and resource use of inpatient and outpatient specialized care [19]. The Cause of Death Register was applied for data on time and cause of death [20]. The Swedish Prescribed Drug Register was used for information on purchased ostomy products and prescribed medication [21]. Two out of the 21 regions in Sweden – Västra Götaland and Jönköping – purchase ostomy products through procurement. As procurement data are not captured by the Swedish Prescribed Drug Register, data from those regions are not included. The Swedish Social Insurance Agency Register provided information on sickness absence of longer than 14 days and its cause as well as early retirement. Primary care data from three Swedish regions were included – Skåne, Östergötland, and Halland – which cover approximately 20% of the Swedish population. Regions were primarily chosen with regards to population size, and with exclusion of the two aforementioned regions with procurement of ostomy products. Furthermore, due to separate data application processes in each region, data from the largest region – Stockholm – was not available for inclusion in the analysis.

Diagnoses were based on The International Statistical Classification of Diseases (ICD-10) codes, while the National Classification of Health Interventions (KVÅ; Swedish acronym for Klassifikation av vårdåtgärder [22]) codes and the Anatomical Therapeutic Chemical (ATC) codes were used respectively for procedures as well as medication and ostomy products prescribed (Table S2).

### Study population

The study population consisted of persons aged 18 years or older with an ICD-10 code for creation of a colostomy, ileostomy, or urostomy, and with at least one ATC code for purchase of ostomy products. The analyses were performed using an incident approach, starting at the index date, which was defined as the date of ostomy creation. The earliest possible index date was 1 January 2006, and persons were followed up to ten years, until death, reversal of a temporary ostomy, termination of purchases of ostomy products (indicating that the ostomy was reversed, or the person had moved to a special care facility with products dispensed directly), or end of study, which was 31 December 2019. Persons were excluded if they had multiple ostomies or died at the index date. Reversal of ostomies was determined from the respective KVÅ codes. Termination of purchases of ostomy products was defined as absence of purchase of ostomy products during a six-month period with a termination date set to three months after the last purchase. This is in accordance with the Swedish reimbursement system, where persons living with an ostomy can purchase ostomy products and drugs for a three-month period.

Three case groups were constructed based on ostomy type: colostomy, ileostomy, and urostomy. Each person was matched with two controls from the general population, identified by Statistics Sweden and based on age, gender, and region. Matched controls were indexed and followed for the same time as their respective cases.

### Definition of outcomes

The resource utilization analysis consisted of the number of healthcare contacts, visits, and admissions per person. Unit cost for healthcare resource utilization were derived from retrospective Diagnose Related Group (DRG) lists and base tariff for 2019 [23], which is a common approach in patient registry-based studies [24]. DRGs refer to a clinically coherent set of patients, for which resource demand and the associated costs experienced by the hospitals is defined. The weight for a specific DRG is calculated as the cost relative to an average patient (all patients, all DRGs), for which the cost-weight is set to 1. In the national DRG-system in Sweden for 2019, a cost-weight = 1 was 57,469 SEK. The strength of DRG-based costing is the transferability of resource utilization and associated costs across different time periods and settings. For costing of primary care contacts, a stringent approach was chosen to apply to all regions despite different coding practices. The approach was based on most frequently reported variable across regions. Primarily, contact type was costed by relevant DRG codes. If contact type was lacking, any reported DRG code was used, and in lack of either contact type or DRG code, actual reported cost were used. Mean cost was estimated per person based on all individuals included in the specific comparison. The average cost for primary care was based on the individuals from the 3 regions from which primary care data were obtained. The healthcare cost covered the following categories: inpatient care, specialized outpatient care (care in specialist areas), emergency care, primary care, home care, ostomy products, and prescribed medication. The 20 most common reasons (primary and secondary diagnosis) for 30-day readmission after ostomy creation starting from a minimum of one day following discharge, hospitalization during the first year, and hospitalization during the entire study period were established based on the occurrence of ICD-10 codes. The number of visiting persons with a specific diagnosis was lower than the number of visits with that specific diagnosis, since a person may be registered for more than one diagnosis at a visit.

The indirect healthcare cost included costs of sickness absence and early retirement costed by the human capital approach (based on the average salary for 2019 including labor market contributions [25, 26]). Cost of sickness absence was set to 80% of the salary and included the first 14 days paid by the employer in accordance with Swedish legislation [27].

The healthcare resource utilization and direct and indirect costs were presented per person for the index admission; year 1 (excl. index admission); year 2; and as the annual averages of years 3–5 and years 6–10. Cost per person was presented in SEK (Swedish krona; 100 SEK was equivalent to 9.58 EUR per 31 December 2019).

### Statistical analysis

Categorical variables are described as absolute number and percentage. Continuous variables are described with mean and standard deviation (SD) and comparison between case and matched control groups was performed with Mann-Whitney U test. Statistical significance was based on group medians while means are presented as they are required for costing analyses. Weighted average was used to describe results across the three ostomy groups. To compute annual estimates, the mean daily resource utilization and cost per person were calculated and multiplied by 365. Statistical significance of differences in resource utilization and cost between case and control was assessed on actual numbers during the full analysis periods. A *p* value < 0.05 was considered statistically significant. Analyses were performed in SAS version 9.4 (SAS Institute, Cary, NC, USA).

## Results

### Demographics

In total, 40,988 cases were identified during the study period: 19,645 (47.9%) had a colostomy created: 16,408 (40.0%), an ileostomy; and 4,935 (12.0%), a urostomy (Table 1). The underlying diagnoses for colostomy and ileostomy creations were primarily bowel cancer (50.0% and 55.8% respectively), with IBD accounting for an additional 20.6% of ileostomies. The underlying cause for urostomy creation was mainly bladder cancer (85.0%). Other underlying diagnoses were not defined. The majority of persons with colostomy and urostomy were over 70 years of age at ostomy creation, while those with an ileostomy were about 7 years younger on average. In the colostomy group there were slightly fewer males than females (46.5% males vs. 53.5% females) while the opposite was true for the ileostomy group (53.4% males vs. 46.6% females). In the urostomy group the male/female ratio was 69.7%/30.3%. There was no difference observed in the prevalence of diabetes (around 12%) and hypertension (approximately 27%) between cases and controls. For the colostomy group, 85.5% of the ostomies were permanent, in contrast to the ileostomy group where 50.4% were permanent. For those with a temporary ostomy, the mean reversal time was below one year. The urostomies were permanent in all cases. The death rate was high for all case groups compared to control groups: 19.9% in the colostomy group died during the first year and 13.7% the second year, and in the ileostomy and urostomy groups 14.4% died during the first year and 9.4% and 10.4% respectively during the second year.

**Table 1:** Demographics and pertinent clinical characteristics of case and control groups at index

	Colostomy		Ileostomy		Urostomy	
	Case	Control	Case	Control	Case	Control
	n = 19,645	n = 38,634	n = 16,408	n = 32,517	n = 4935	n = 9775
Underlying diagnosis (12 months before index), n (%)						
Bowel cancer	9827 (50.0%)	99 (0.3%)	9152 (55.8%)	70 (0.2%)	120 (2.4%)	22 (0.2%)
Bladder cancer	252 (1.3%)	164 (0.4%)	138 (0.8%)	86 (0.3%)	4197 (85.0%)	53 (0.5%)
IBD	498 (2.5%)	154 (0.4%)	3379 (20.6%)	147 (0.5%)	43 (0.9%)	50 (0.5%)
Age, n (%)						
-20 years	7 (0.0%)	14 (0.0%)	46 (0.3%)	92 (0.3%)	1 (0.0%)	2 (0.0%)
20–29 years	184 (0.9%)	368 (1.0%)	752 (4.6%)	1504 (4.6%)	13 (0.3%)	26 (0.3%)
30–39 years	326 (1.7%)	652 (1.7%)	804 (4.9%)	1608 (5.0%)	33 (0.7%)	66 (0.7%)
40–49 years	909 (4.6%)	1816 (4.7%)	1288 (7.9%)	2575 (7.9%)	102 (2.1%)	204 (2.1%)
50–59 years	2072 (10.6%)	4142 (10.7%)	2349 (14.4%)	4695 (14.5%)	393 (8.0%)	785 (8.0%)
60–69 years	4526 (23.0%)	9017 (23.3%)	4461 (27.3%)	8881 (27.4%)	1455 (29.5%)	2898 (29.6%)
70–79 years	6079 (31.0%)	12,033 (31.2%)	4573 (28.0%)	9047 (27.9%)	2349 (47.6%)	4652 (47.6%)
80–89 years	4728 (24.1%)	9109 (23.6%)	1859 (11.4%)	3601 (11.1%)	587 (11.9%)	1138 (11.6%)
90- years	806 (4.1%)	1467 (3.8%)	226 (1.4%)	414 (1.3%)	2 (0.0%)	4 (0.0%)
Age at ostomy creation, mean ± SD	70.7 ± 13.4	70.5 ± 13.3	63.4 ± 16.0	63.2 ± 15.9	70.2 ± 9.1	70.1 ± 9.1
Gender, n (%)						
Male	9141 (46.5%)	17,958 (46.5%)	8761 (53.4%)	17,349 (53.4%)	3441 (69.7%)	6813 (69.7%)
Female	10,504 (53.5%)	20,676 (53.5%)	7647 (46.6%)	15,168 (46.6%)	1494 (30.3%)	2962 (30.3%)
Comorbidities, n (%)						
Diabetes	2615 (13.3%)	4451 (11.5%)	2054 (12.5%)	3408 (10.5%)	751 (15.2%)	1353 (13.8%)
Hypertension	5626 (28.6%)	10,370 (26.8%)	4266 (26.0%)	7837 (24.1%)	1589 (32.2%)	3067 (31.4%)
Ostomy characteristics, n (%)						
Permanent	16,872 (85.9%)	NA	8264 (50.4%)	NA	NA	NA
Temporary	2772 (14.1%)	NA	8144 (49.6%)	NA	NA	NA
Months to reversal, mean ± SD	11.5 ± 9.3	NA	9.40 ± 8.37	NA	NA	NA
Death in study period (exclusive death during ostomy surgery), n (%)						
During the first year	3909 (19.9%)	1081 (2.8%)	2360 (14.4%)	371 (1.1%)	711 (14.4%)	204 (2.1%)
During the second year	1606 (13.7%)	640 (2.9%)	589 (9.4%)	143 (1.2%)	387 (10.4%)	123 (1.7%)

IBD: inflammatory bowel disease; SD: standard deviation

### Direct healthcare utilization and costs

#### Hospital admissions

Surgery for the creation of an ostomy (index admission) required on average (weighted) 15.2 days in hospital and the associated costs were 177,400 SEK per person (Table 2). A colostomy was the most costly ostomy to create, while creation of a urostomy was the least costly.

**Table 2:** Mean healthcare resource utilization and cost per person in SEK at index admission

	Colostomy	Ileostomy	Urostomy
Index admission (days), mean ± SD)	14.9 ± 12.7	15.3 ± 13.9	15.8 ± 15.1
Index cost (1000 SEK), mean ± SD)	174.2 ± 86.5	184.2 ± 88.1	167.5 ± 60.3

SD: Standard deviation; SEK: Swedish krona

**Fig. 1:**
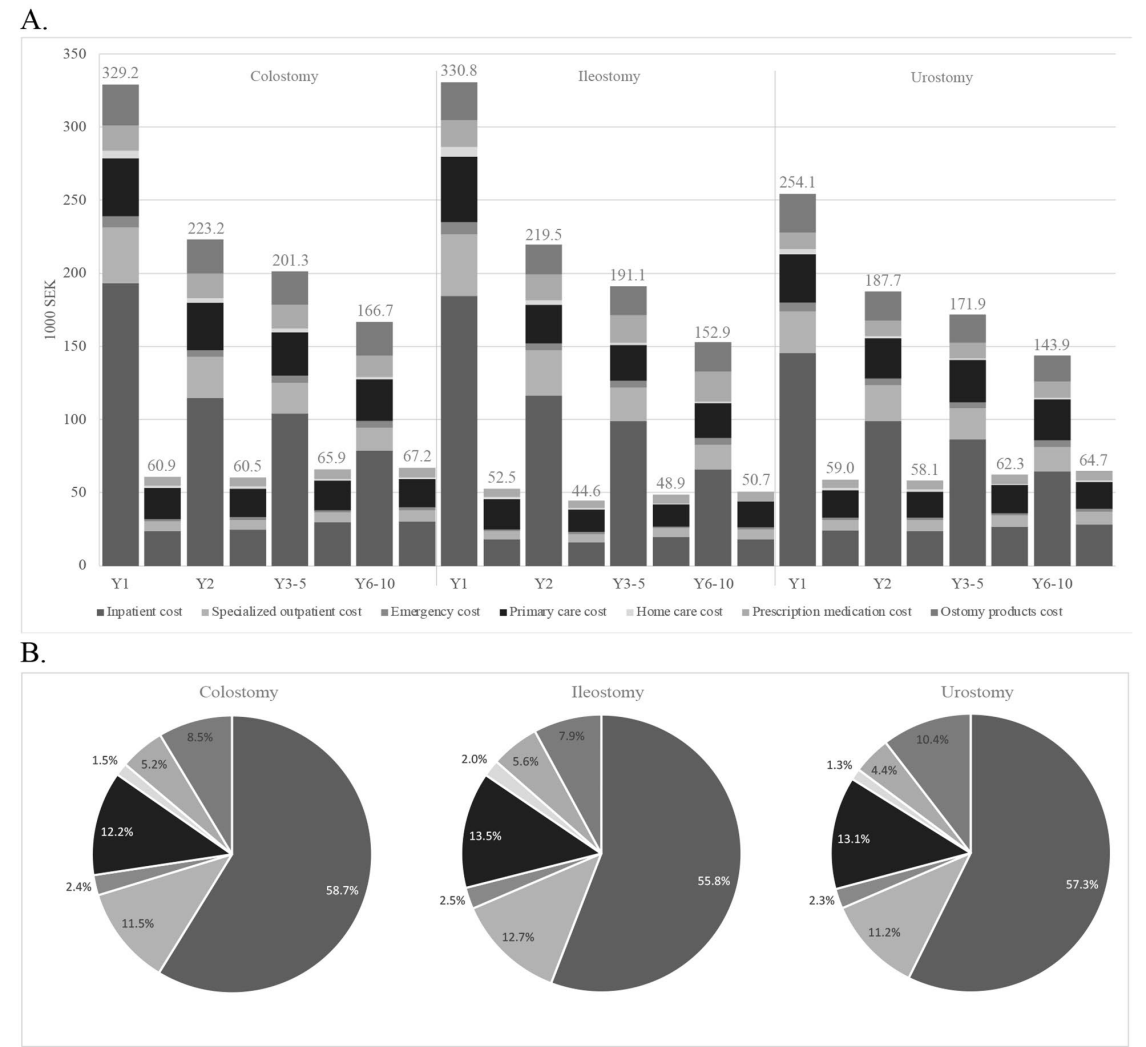
**A**. Mean annual direct healthcare cost per person after ostomy creation presented in 1000 SEK. **B**. Relative distribution of healthcare categories for case groups in year 1 For a given follow-up time point, the first bar refers to the case group and the second bar to the matched control group Mann-Whitney U test was used to test for difference between case and controls. Statistical significance was based on group medians while means are presented as they are required for costing analysis. All differences between case and control groups are significant, *p* < 0.0001. SEK: Swedish krona; Y: year. Please refer to Table S3 for mean and standard deviation

Statistically significant differences in number of hospital admissions and associated costs were consistently demonstrated between ostomy groups and their controls (*p* < 0.0001). In the first year (excluding index admission), a person with an ostomy on average visited the hospital 2.9 times and stayed for a total of 23.0 days (respectively 8 and 11 times higher than controls) (Table 3). In years 6–10, this decreased to 1.2 visits and 8.1 days annually (both 3 times higher than controls). The cost of hospital admissions accounted for 193,300, 184,700, and 145,600 SEK per person in the first year (8, 10, and 6 times higher than controls) for colostomy, ileostomy, and urostomy respectively decreasing to 78,500, 65,800, and 64,500 SEK (3, 4, and 2 times higher than controls) annually in years 6–10. The cost of hospital admission was the main driver of the total direct healthcare cost for all ostomy groups, constituting on average 57.4% in the first year (Fig. 1).

**Table 3:** Mean annual healthcare resource utilization per person after ostomy creation

	Colostomy		Ileostomy		Urostomy	
	Case	Control	Case	Control	Case	Control
Hospital admissions (visits per year), mean ± SD						
Y1	2.9 ± 4.3*	0.4 ± 2.0	3.1 ± 4.6*	0.3 ± 1.7	2.6 ± 3.7*	0.4 ± 2.1
Y2	1.9 ± 5.4*	0.4 ± 2.4	1.8 ± 4.3*	0.3 ± 1.5	1.7 ± 3.8*	0.4 ± 1.9
Y3-5	1.7 ± 4.2*	0.5 ± 2.0	1.6 ± 3.7*	0.3 ± 1.2	1.4 ± 3.7*	0.4 ± 1.6
Y6-10	1.4 ± 3.2*	0.5 ± 1.9	1.1 ± 2.5*	0.3 ± 0.9	1.1 ± 2.7*	0.5 ± 1.3
Hospital admissions (days per year), mean ± SD						
Y1	24.8 ± 46.0*	2.5 ± 13.5	24.0 ± 47.2*	1.7 ± 16.5	20.2 ± 37.8*	2.3 ± 12.9
Y2	13.6 ± 43.7*	2.6 ± 17.5	11.6 ± 33.5*	1.4 ± 10.0	11.9 ± 34.3*	2.2 ± 14.0
Y3-5	12.0 ± 31.7*	2.8 ± 12.9	9.9 ± 26.5*	2.0 ± 11.1	10.2 ± 30.2*	2.6 ± 10.9
Y6-10	9.6 ± 32.0*	3.2 ± 13.8	6.7 ± 20.7*	1.7 ± 7.2	7.1 ± 25.2*	2.8 ± 11.5
Specialized outpatient visits (visits per year), mean ± SD						
Y1	8.3 ± 10.7*	1.5 ± 4.5	9.2 ± 9.9*	1.3 ± 3.9	6.1 ± 7.5*	1.6 ± 5.2
Y2	5.7 ± 10.7*	1.5 ± 5.0	6.7 ± 11.6*	1.3 ± 4.4	5.2 ± 8.7*	1.7 ± 5.9
Y3-5	4.6 ± 10.2*	1.6 ± 3.8	5.0 ± 9.1*	1.4 ± 4.5	4.6 ± 9.0*	1.7 ± 4.0
Y6-10	3.5 ± 9.4*	1.7 ± 4.8	3.7 ± 8.1*	1.6 ± 5.4	3.7 ± 9.1*	2.0 ± 3.5
Primary care excl. home care (contacts per year), mean ± SD						
Y1	22.1 ± 24.3*	11.8 ± 15.3	23.3 ± 27.6*	11.5 ± 15.0	17.9 ± 20.1*	10.8 ± 15.5
Y2	17.3 ± 23.5*	10.9 ± 15.4	14.2 ± 21.6*	8.8 ± 15.9	14.4 ± 20.0*	10.2 ± 16.2
Y3-5	16.0 ± 20.9*	11.2 ± 15.1	13.2 ± 18.6*	8.4 ± 12.5	15.3 ± 20.1*	11.0 ± 16.2
Y6-10	15.7 ± 18.8*	10.9 ± 14.6	13.2 ± 16.6*	9.6 ± 17.3	16.1 ± 18.6*	10.5 + 15.6

Mann-Whitney U test was used to test for difference between case and controls. Statistical significance was based on group medians while means were presented as they are required for costing analyses. SD: Standard deviation; Y: year; *: *p* < 0.0001

Besides complications due to the underlying disease, complications related to the ostomy contributed to the high utilization and derived cost of hospital admissions. In total, 19.7% of the colostomy group (3,773 visits), 25.9% of the ileostomy group (16,408 visits), and 22.2% of the urostomy group (4,935 visits) were readmitted to hospital within 30 days after ostomy creation (Table S4-S6). The artificial opening was responsible for 19.3–22.8% of total number of 30-day readmission visits, 14.4–15.8% of hospital admissions during the first year after ostomy creation, and 19.7–21.4% of hospital admission during the entire study period. For the ileostomy group, dehydration was furthermore responsible for 13.0% of 30-day readmission visits, while hospital admissions due to dehydration constituted 4.7% of the total number of visits during the first year after ostomy creation and remained on this level when analyzing the entire study period (4.5%).

#### Specialized outpatient visits

The number of specialized outpatient visits per person and the associated costs were at any time point significantly different between ostomy groups and their controls (*p* < 0.0001). The number of specialized outpatient visits was on average 8.4 in the first year for a person with an ostomy (6 times higher than controls), which decreased to 3.6 annually in years 6–10 (2 times higher than controls) (Table 3). The cost of specialized outpatient visits for a person with an ostomy accounted for on average 11.9% of total direct healthcare cost in the first year (Fig. 1). It amounted to on average 38,400 SEK per person in the first year (6 times higher than controls) and declined to on average 16,400 SEK per person annually at years 6–10 (2 times higher than controls). The cost of specialized outpatient visits was lowest for a person with urostomy.

#### Primary care

The number of contacts with primary care per person and the associated costs were significantly different between the ostomy groups and their matched controls for all years (*p* < 0.0001). Primary care was contacted on average 22.1 times per person in the first year (2 times higher than controls), declining to 14.7 times per person annually in years 6–10 (1.4 times higher than controls) (Table 3). The cost per person of primary care constituted on average 12.8% of the total direct healthcare cost in the first year and averaged 41,100 SEK per person with ostomy (2 times higher than controls) (Fig. 1). The cost of primary care for a person with urostomy was lower than for persons from the two other groups in the first year. In years 6–10, the cost had decreased to an annual level, which was quite similar for all three ostomy groups: an average of 26,700 SEK per person (1.4 times higher than controls).

#### Ostomy products

**Fig. 2:**
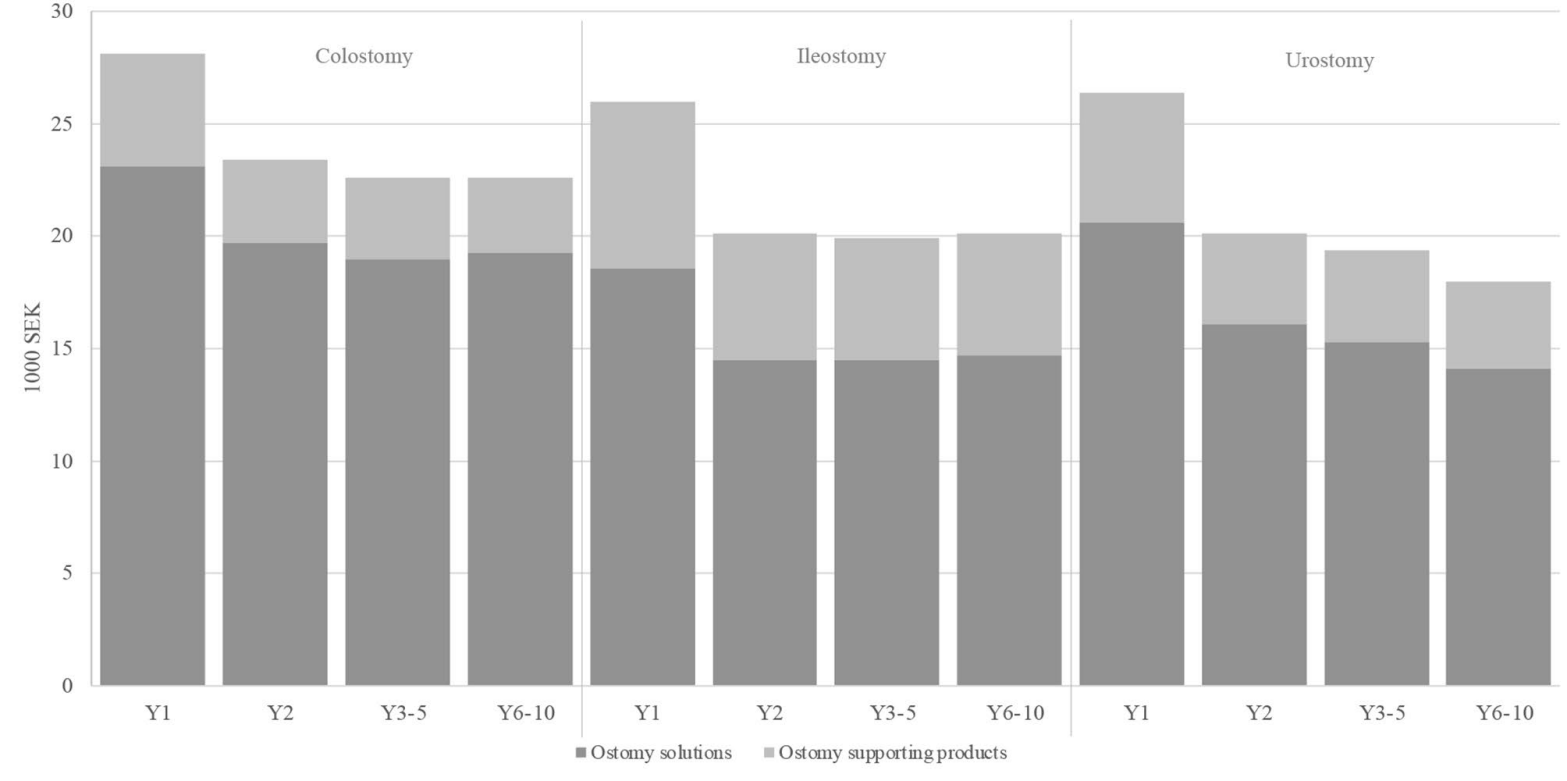
Mean annual cost per person of ostomy products and supporting products presented in 1000 SEK. SEK: Swedish krona; Y: year. Please refer to Table S7 for mean and standard deviation

The cost per person for ostomy solutions and supporting products (accessories) accounted for 8.5% of the total direct healthcare cost (Fig. 1), which amounted to 27,100 SEK per person on average for all three ostomy types in the first year (Fig. 2). In year 2 and for the rest of the study period, the total product-related cost declined to on average 21,300 SEK per person. The cost of ostomy solutions was highest for a person with colostomy, while the cost of supporting products was highest for a person with ileostomy throughout the entire study.

#### Prescribed medication

The cost per person of total prescribed medication constituted on average 17,000 SEK in the first year across the three subgroups (3 times higher than controls) and was equivalent to 5.3% of the total direct healthcare cost (Fig. 1). The cost of prescription medication for a person with urostomy was the lowest of the three ostomy types. In general, the cost of prescription medication remained on the initial level throughout the entire study period. In relation to prescription medication, which – among other treatment indications – are used to treat ostomy-related complications, it is noteworthy that the annual cost per person was very high for pain medication irrespective of ostomy type, although it was highest for persons with a colostomy (Table 4). In contrast, for a person with ileostomy the costs of prescription medication for bowel dysfunction problems were higher than for the other two ostomy groups. The cost per person of total and selected prescribed medication was significantly different between ostomy groups and their controls during the entire study (*p* < 0.0001).

**Table 4:** Mean annual costs per person in SEK of selected medications used to treat ostomy-related complications

		Skin complications	Bowel dysfunction	Pain	Mood disorders	Infections
Colostomy, mean ± SD						
Y1	Case	523 ± 3269*	492 ± 869*	1930 ± 7720*	461 ± 1299*	80 ± 1720*
	Control	134 ± 933	79 ± 358	187 ± 1706	237 ± 950	7 ± 483
Y2	Case	555 ± 6532*	395 ± 1108*	1750 ± 18,310*	468 ± 1443*	82 ± 4010*
	Control	143 ± 2132	85 ± 376	182 ± 1480	239 ± 846	16 ± 1940
Y3-5	Case	454 ± 4687*	387 ± 1021*	1420 ± 6560*	482 ± 1276*	97 ± 4143*
	Control	139 ± 1263	90 ± 430	199 ± 1300	240 ± 763	13 ± 943
Y6-10	Case	352 ± 1769*	355 ± 1058*	929 ± 4548*	509 ± 1807*	13 ± 214*
	Control	132 ± 773	89 ± 280	211 ± 2080	252 ± 733	14 ± 662
Ileostomy, mean ± SD						
Y1	Case	522 ± 4739*	770 ± 2565*	1340 ± 5690*	358 ± 1357*	125 ± 2588*
	Control	118 ± 1374	55 ± 338	118 ± 939	189 ± 856	11 ± 1155
Y2	Case	432 ± 4947*	626 ± 3368*	1160 ± 6360*	349 ± 1185*	93 ± 3878*
	Control	107 ± 912	56 ± 402	143 ± 1270	182 ± 844	8 ± 452
Y3-5	Case	586 ± 11,732*	565 ± 2277*	1030 ± 5040*	402 ± 1247*	69 ± 1859*
	Control	115 ± 846	59 ± 352	149 ± 1034	176 ± 685	3 ± 69
Y6-10	Case	304 ± 1775*	462 ± 2300*	643 ± 3286*	433 ± 1156*	31 ± 827*
	Control	101 ± 387	53 ± 290	146 ± 1265	177 ± 648	2 ± 33
Urostomy, mean ± SD						
Y1	Case	477 ± 2357*	348 ± 616*	900 ± 3828*	301 ± 833*	79 ± 1995*
	Control	131 ± 1450	68 ± 310	169 ± 3718	193 ± 755	3 ± 76
Y2	Case	314 ± 1522*	255 ± 908*	983 ± 4556*	314 ± 1180*	30 ± 931*
	Control	105 ± 353	68 ± 305	137 ± 1082	189 ± 793	3 ± 83
Y3-5	Case	302 ± 1011*	258 ± 1174*	770 ± 3796*	300 ± 860*	13 ± 333*
	Control	123 ± 756	69 ± 241	193 ± 3051	200 ± 689	11 ± 636
Y6-10	Case	495 ± 7271*	261 ± 1000*	409 ± 1600*	350 ± 988*	196 ± 7187*
	Control	192 ± 2736	86 ± 413	139 ± 729	202 ± 615	70 ± 2640

Mann-Whitney U test was used to test for difference between case and controls. Statistical significance was based on group medians while means are presented as they are required for costing. SD: standard deviation; SEK: Swedish krona; Y: year; *: *p* < 0.0001

#### Total direct healthcare cost (excl. Index admission)

The cost per person of the total annual healthcare was significantly higher for the three ostomy groups compared to their matched control groups at all time points (*p* < 0.0001). However, the cost difference declined over time. In the first year, the total healthcare cost per person amounted to 329,200 SEK for colostomy, 330,800 SEK for ileostomy, and 254,100 SEK for urostomy, which was equivalent to 5, 6, and 4 times higher costs than for the matched controls (Fig. 1). In years 6–10, the annual healthcare cost per person for the three ostomy types decreased to 166,700 SEK, 152,900 SEK, and 143,900 SEK (2–3 times higher than controls).

### Indirect costs

**Fig. 3:**
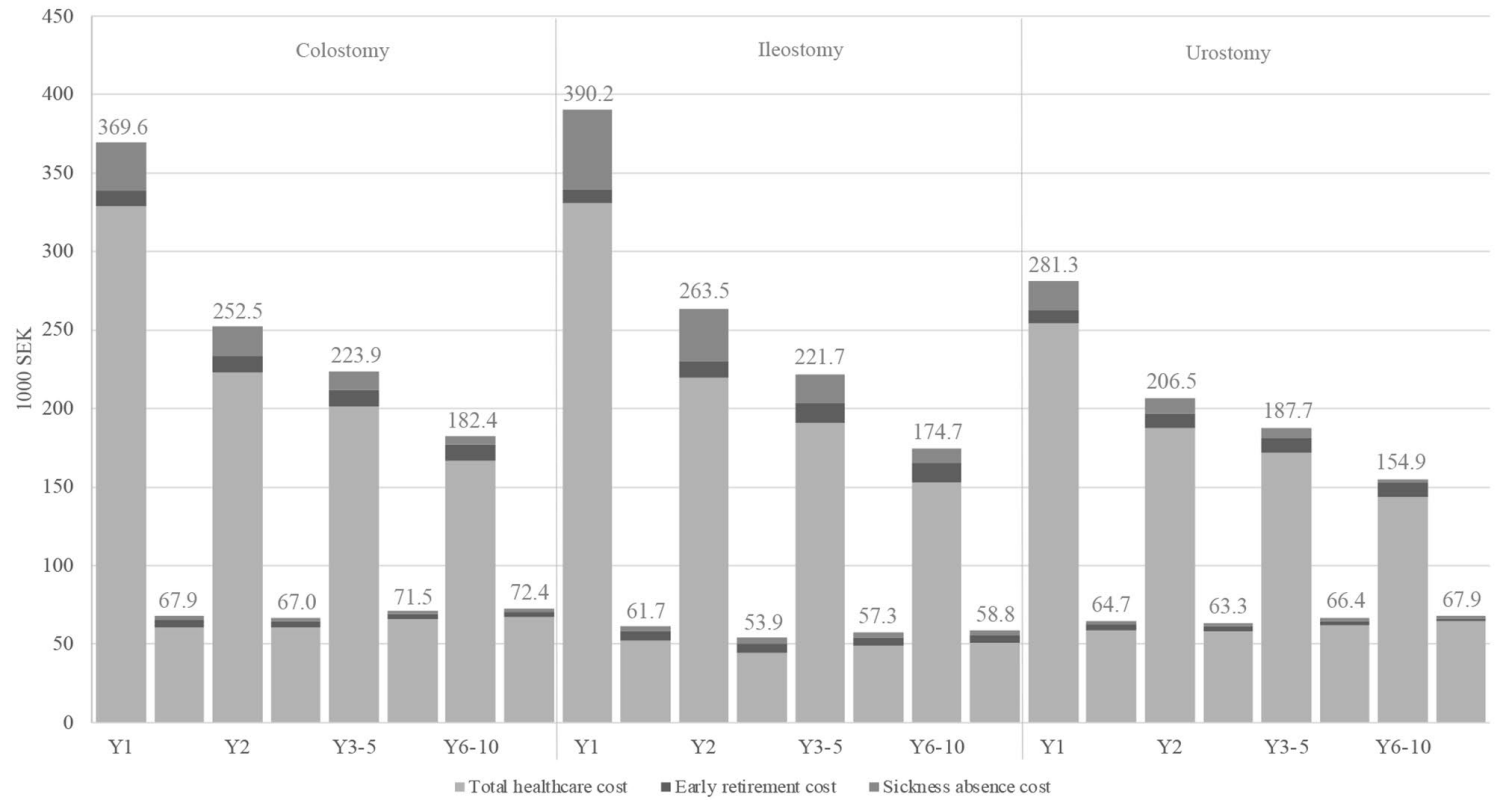
Mean annual direct and indirect cost per person presented in 1000 SEK. For a given follow-up time point, the first bar refers to the case group and the second bar to the matched control group. Mann-Whitney U test was used to test for difference between case and controls. Statistical significance was based on group medians while means are presented as they are required for costing analyses. All differences between case and control groups are significant, *p* < 0.0001. SEK: Swedish krona; Y: year. Please refer to Table S8 for mean and standard deviation

The annual indirect cost per person, which consisted of costs relating to sickness absence of more than 14 days and early retirement, were significantly higher for ostomy groups compared to matched controls at all time points (*p* < 0.001), except sickness absence during years 6–10 for urostomy. On average, the annual indirect cost reached 9,100 SEK per person for early retirement (2 times higher than controls) and 37,400 SEK per person for sickness absence (14 times higher than controls) in the first year (Fig. 3). The annual cost of early retirement increased to 11,200 SEK per person (3 times higher than controls) in years 6–10, and sickness pay decreased to 6,400 SEK per person (2 times higher than controls). In general, the cost of sickness absence decreased considerably over time and was highest for the ileostomy group during the entire study period. The expenses of early retirement and sickness absence were relatively small compared to the total direct healthcare cost, corresponding to an average of 12.5% of the sum of direct and indirect cost. The annual total direct and indirect healthcare cost amounted to 369,600 SEK for a person with a colostomy, 390,200 SEK for ileostomy, and 281,300 SEK for urostomy in the first year after ostomy creation.

## Discussion

This is the first Swedish registry-based nationwide study estimating the healthcare resource utilization and direct and indirect costs up to ten years after creation of a colostomy, ileostomy, or urostomy due to various diseases. The annual utilization and cost of healthcare resources were significantly elevated compared to matched controls up to 10 years after ostomy creation, although the elevation became less pronounced over time. The direct healthcare cost was primarily driven by hospitalization, which in turn was impacted by complications to the ostomy as well as the underlying disease.

The high total direct healthcare cost in our study reflected the fact that 67.4% of the underlying diseases, which required an ostomy creation, were bowel and bladder cancer as well as IBD, which are resource-intensive diseases to treat [28, 29]. The total healthcare cost has been shown to be highest in the first year after cancer diagnosis, and the majority of these costs were recorded as being cancer-specific [30]. Additionally, management and treatment of ostomy-related complications have been shown to also have a considerable impact on the use of healthcare resources and the associated costs [1, 12–15]. In our study, the mortality rate was high within the first years, especially among the colostomy group. This could be due to the fact that the persons in this group were relatively old, potentially sicker, and therefore at a higher risk of late-stage cancers and death.

In accordance with our study, a Danish registry study [16] and two studies based on German claims data [17, 18] demonstrated that people living with a permanent ostomy incurred a significantly higher economic burden on the healthcare system compared to matched controls [16, 17], as did people with a newly created ostomy compared to the year before their ostomy creation [18]. As in our study, hospitalizations were by far the main cost driver in these studies.

Our study indicated that a contributing factor for the increased cost of hospitalization in the first year after ostomy creation was frequent readmissions within 30 days after the ostomy creation. Even though complications from the underlying diseases were responsible for the majority of readmissions and hospitalizations, complications related to the ostomy were also common reasons. This is in line with previous evidence, which demonstrated that readmissions following ostomy creation are both common and expensive [12, 31, 32]. In our study, dehydration resulted in 13.0% of the total number of 30-day readmissions after creation of an ileostomy. A 30-day readmission incidence of 5.0% after ileostomy surgery has previously been presented in a systematic review by Liu et al. [32]. However, this discrepancy could be due to differences in prevalence of underlying diseases as dehydration is more common with IBD than with bowel cancer [31, 32].

A striking finding in our study was the elevated cost of prescribed pain medication. Whilst this emphasized that these persons were suffering disproportionately, it was not possible to conclude if the pain was a cause of the underlying disease or related to the ostomy. The high cost of medication for bowel dysfunction for persons with ileostomy may reflect that these persons often experience periods with high stoma output [33].

The cost of ostomy products remained on an almost constant level after a slightly more expensive first year. This supports research which has illustrated that in the first period after ostomy creation, many different ostomy products are tried out before the most appropriate product is selected. During this period the use of supporting products such as rings, seals, paste, tape, belt, and powder increases concurrently, which in turn increases the cost [13–15, 34–36].

Our results also revealed that in the first year after ostomy creation, the income transfer payments primarily consisted of cost of sickness absence, which gradually changed to cost of early retirement during the following years. The cost of sickness absence and retirement per person with an ostomy was relatively low and was likely a consequence of a high average age, at which the persons would likely be transitioning to age pension. This explanation seemed to fit with the finding that cost of sickness absence and early retirement were highest for the ileostomy group, which had the lowest average age. This suggests that the creation of an ostomy impacted the ability of the relatively younger members of this group to continue to participate in the work force, as might otherwise be expected at their age. This finding confirms conclusions from previous studies, that living with an ostomy influences the ability to work, thereby causing partial or complete loss of work [13, 17, 34, 37, 38]. As the cost of productivity loss due to sickness are seldom fully compensated by income transfer payments, it indicates that the impact of living with an ostomy, and particularly an ileostomy, may be significant to the personal income level as well [39].

The hospitalization cost in this study were very high and to some degree driven by ostomy-related complications, which are partly avoidable. Proper marking and surgical construction of the ostomy as well as cautious follow-up are important to reduce the incidence of complications [9]. This calls for cautious surgical procedures as well as improvements regarding pre-operative and post-operative ostomy education to prepare for discharge and increase the ostomy-related support after discharge [31]. The use of dedicated stoma care nurses after discharge has been shown to decrease the incidence of ostomy-related complications, thereby reducing the costs [5, 7]. Likewise, patient education regarding ostomy management has shown to reduce readmission rates, length of readmissions, as well as associated costs [40, 41]. Moreover, a recent scoping review suggests that strenghtening the role of stoma care nurses could impact the healthcare system positively by increasing the quality of healthcare services provided and the quality of life for the persons living with an ostomy [42]. Lastly, a proper fit between the peristomal body profile and the ostomy product(s) guided by a stoma care nurse has been shown to reduce the number of leakages and in turn reduce the incidence of PSCs [43, 44].

### Strengths and limitations

The strengths of this study include the use of data from nationwide registries to ensure a real-world representative sample of people living with an ostomy in Sweden and to minimize the risk of information and selection bias. The large study population reduced the risk of random variations in the estimates of healthcare utilization and cost. In addition, the inclusion of matched control groups increased the power and reduced the bias of the results. The inclusion of income transfer payment is a fundamental advantage of our methodology, as this reflects a part of the indirect impact for both person and society. Lastly, the longitudinal study design made it possible to evaluate the outcomes over different lengths of time. Regarding limitations, the data quality of the registries is dependent on correct diagnose coding into the registries. However, the registries are considered valid and are used as background data for political and economic decisions in Swedish healthcare policies. Data on ostomy products were based on data from 19 out of 21 regions and data from primary care were only included for three out of 21 regions; however, we believe that it is representative for all regions. When persons are discharged from the hospital it varies between regions if they are followed up in primary care or by a stoma care nurse and this cannot be interpreted from the registries as nurse visits are not registered. Our estimates of productivity loss were based on a conservative approach, which only included sickness absence and early retirement, which means that the actual cost is likely higher. Taken together, our estimates of the healthcare resource utilization and associated cost is likely conservative. The nature of the study was observational, and causality cannot be inferred. Despite these limitations, this study adds details to the current understanding of the burden of living with an ostomy.

## Conclusions

This nationwide study reported that persons with an ostomy were burdened by complications related to the ostomy in addition to the underlying disease. This had a substantial impact on the healthcare cost for at least ten years after ostomy creation and it was driven primarily by hospitalization. In addition, working ability seemed to be negatively impacted, indicated by increased sickness pay and early retirement pension. The study therefore calls for improved management and support of ostomy care for the benefit of the affected persons and for the cost of society. Chart review studies are recommended to estimate the economic impact of cost elements, which are not captured by registries, such as visits to stoma care nurse.

### Electronic supplementary material

Below is the link to the electronic supplementary material.

Supplementary Material 1

## Data Availability

The data which support the findings of this study are available from the National Board of Health and Welfare, the Swedish Social Insurance Agency, Statistics Sweden, Region Östergötland, Region Skåne, and Region Halland, but restrictions apply to the availability of these data, which were used under license for the current study, so which are therefore not publicly available. However, data are available from the author Annabelle Forsmark upon reasonable request and with permission of the National Board of Health and Welfare, the Swedish Social Insurance Agency, Statistics Sweden, Region Östergötland, Region Skåne, and Region Halland.

## Abbreviations

IBD: Inflammatory bowel disease
PSCs: Peristomal skin complications
ICD-10: International Statistical Classification of Diseases
KVÅ: National Classification of Health Interventions (Swedish acronym for Klassifikation av vårdåtgärder)
ATC: Anatomical Therapeutic Chemical
DRG: Diagnose Related Group
SEK: Swedish krona
SD: Standard deviation
