# Supplementary material for: Short- and long-term direct and indirect costs of illness after ostomy creation – a Swedish nationwide registry study
Source: BMC Health Serv Res. 2023 Aug 8;23:837. doi: 10.1186/s12913-023-09850-5 (PMC10408161; doi:10.1186/s12913-023-09850-5)
Supplement: Supplementary file 1 — Supplementary Material 1 [file 12913_2023_9850_MOESM1_ESM.docx]

**Supplementary**

Table S1 Data sources applied.

| **Data source** | **Description** | **Data obtained** |
| --- | --- | --- |
| Swedish Prescribed Drug Register | Nationwide register on all prescribed drugs purchased in pharmacies, irrespective of reimbursement status. Started in July 2005. | Patient identification  Stoma appliances  Pharmaceutical use  Pharmaceutical costs |
| National Patient Register | Nationwide register on all inpatient care in Sweden, and, from 2001, also outpatient specialist visits. | Patient demographics  Clinical outcomes  Healthcare resource use  Healthcare costs |
| Cause of Death Register | Nationwide register on time and cause of death | Time of death  Cause of death |
| Register of Swedish Social Insurance Agency | Nationwide register on sickness absence periods lasting longer than 14 days, including information on diagnosis (first 3 signs in the ICD-10 code). | Sickness absence and cause  Early retirement |
| Swedish Longitudinal Integrated Database for Health Insurance and Labour Market Studies | Nationwide register on socioeconomic data | Unemployment  Disposable income |
| Regional health care registers | Regional registers on health care resource use. Data details and time horizons differ by region. Data from the regions Skåne, Östergötland, and Halland were included. | Clinical outcomes  Primary healthcare resource use  Primary healthcare costs |

Table S2 Codes for applied diagnoses, procedures, prescription medications, and ostomy products.

|  | Codes | Comment |
| --- | --- | --- |
| Ostomy creation | | |
| Colostomy | JGB10, JGB11, JFF23, JFF24, JFF26, JFF27, JFF30, JFF31, JFB60, JFB61, JFB63, JFB64 | KVÅ codes |
|  | Z43.3, Z93.3 | ICD-10 codes |
| Ileostomy | JFH10, JFH11, JFH20, JFH21, JFF13, JFF16, JGB40 | KVÅ codes |
|  | Z43.2, Z93.2 | ICD-10 codes |
| Urostomy | KBJ00, KBJ10, KBJ20, KBJ40, KBJ60 | KVÅ codes |
|  | Z43.5, Z43.6, Z93.5, Z93.6 | ICD-10 codes |
| Other kind of ostomy | JFF60, JFF96, JFF97 | KVÅ codes |
|  | Z43.4, Z46.5, Z93.4 | ICD-10 codes |
| Removal of ostomy | | |
| Colostomy | JFG10, JFG30, JFG33, JFG36 | KVÅ codes |
| Ileostomy | JFG00, JFG20, JFG23, JFG26, JFG29 | KVÅ codes |
| Underlying diagnoses 12 months before index | | |
| IBD | K50, K51 | ICD-10 codes |
| Bowel cancer | C17, C18, C19, C20, C21 | ICD-10 codes |
| Bladder cancer | C67 | ICD-10 codes |
| Comorbidities at index | | |
| Hypertension | I10, I11, I12, I13, I15 | ICD-10 codes |
| Diabetes | E10, E11, E12, E13, E14 | ICD-10 codes |
| Selected prescription medication and ostomy products | | |
| Skin complications excl. antibiotics with UTI indication | D01A, D06A, D07A, J01, J02 | ATC codes |
| Bowel dysfunction | A06, A07D | ATC codes |
| Pain | N02A | ATC codes |
| Mood disorders | N05B, N05C, N06A, N06C | ATC codes |
| Infections | J01, J02A | ATC codes |
| Ostomy products | Y90 | ATC codes |
| Reasons for hospitalization | | |
| Diagnosis |  | ICD-10 codes |

Table S3 Mean annual direct healthcare cost per person after ostomy creation presented in 1000 SEK.

|  | | Inpatient cost | Specialized outpatient cost | Emergency cost | Primary care cost | Home care cost | Prescription medication cost | Ostomy products cost^§^ |
| --- | --- | --- | --- | --- | --- | --- | --- | --- |
| Colostomy, mean ± SD | | | | | | | | |
| Y1 | Case | 193.3 ± 315.7* | 37.9 ± 53.3* | 7.7 ± 16.0* | 40.0 ± 49.0* | 4.9 ± 28.6* | 17.2 ± 33.6* | 28.1 |
|  | Control | 23.9 ± 127.5 | 6.3 ± 20.2 | 1.6 ± 8.1 | 21.4 ± 52.2 | 1.6 ± 12.5 | 6.2 ± 21.4 | NA |
| Y2 | Case | 114.7 ± 362.7* | 27.9 ± 185.1* | 4.7 ± 14.6* | 32.9 ± 71.7* | 2.8 ± 22.8* | 16.8 ± 63.5* | 23.4 |
|  | Control | 24.9 ± 161.9 | 6.7 ± 38.1 | 1.6 ± 16.0 | 19.6 ± 29.8 | 1.6 ± 20.6 | 6.1 ± 19.4 | NA |
| Y3-5 | Case | 104.0 ± 300.2* | 21.1 ± 44.1* | 4.9 ± 19.2* | 29.5 ± 43.1* | 2.8 ± 25.7* | 16.4 ± 39.3* | 22.6 |
|  | Control | 29.8 ± 405.5 | 6.7 ± 21.2 | 1.7 ± 5.9 | 20.1 ± 29.6 | 1.2 ± 11.5 | 6.4 ± 20.8 | NA |
| Y6-10 | Case | 78.5 ± 191.5* | 15.9 ± 43.2* | 4.9 ± 14.5* | 28.5 ± 38.1* | 1.1 ± 3.8* | 15.2 ± 51.1* | 22.6 |
|  | Control | 30.6 ± 124.2 | 7.5 ± 21.2 | 2.0 ± 5.2 | 19.5 ± 28.5 | 0.8 ± 4.7 | 6.9 ± 22.7 | NA |
| Ileostomy, mean ± SD | | | | | | | | |
| Y1 | Case | 184.7 ± 304.8* | 41.9 ± 48.1* | 8.3 ± 18.8* | 44.8 ± 60.5* | 6.5 ± 42.0* | 18.6 ± 50.9* | 26.0 |
|  | Control | 18.1 ± 151.1 | 5.6 ± 19.9 | 1.2 ± 5.7 | 20.9 ± 29.6 | 1.1 ± 4.5 | 5.6 ± 30.9 | NA |
| Y2 | Case | 116.2 ± 317.4* | 31.0 ± 87.6* | 4.9 ± 18.6* | 26.5 ± 43.2* | 3.0 ± 37.9* | 17.8 ± 66.4* | 20.0 |
|  | Control | 15.9 ± 98.9 | 5.9 ± 35.9 | 1.2 ± 6.5 | 15.5 ± 30.7 | 0.8 ± 4.4 | 5.3 ± 32.1 | NA |
| Y3-5 | Case | 98.9 ± 278.6* | 22.9 ± 42.2* | 4.6 ± 13.2* | 24.5 ± 38.6* | 1.6 ± 11.7* | 18.7 ± 74.0* | 19.9 |
|  | Control | 19.5 ± 81.1 | 6.0 ± 22.7 | 1.4 ± 4.4 | 15.0 ± 24.3 | 0.8 ± 8.6 | 6.1 ± 42.4 | NA |
| Y6-10 | Case | 65.8 ± 159.9* | 16.9 ± 36.4* | 4.7 ± 11.7* | 24.1 ± 33.9* | 0.6 ± 2.6* | 20.7 ± 196.6* | 20.1 |
|  | Control | 18.2 ± 69.9 | 6.8 ± 23.5 | 1.5 ± 3.8 | 17.4 ± 34.4 | 0.5 ± 3.3 | 6.4 ± 46.4 | NA |
| Urostomy, mean ± SD | | | | | | | | |
| Y1 | Case | 145.6 ± 233.3* | 28.5 ± 34.3* | 5.9 ± 11.4* | 33.3 ± 40.8* | 3.3 ± 26.0* | 11.1 ± 24.5* | 26.4 |
|  | Control | 24.4 ± 152.5 | 7.0 ± 22.8 | 1.3 ± 5.6 | 19.2 ± 30.5 | 1.1 ± 4.4 | 5.9 ± 20.5 | NA |
| Y2 | Case | 99.2 ± 236.4* | 24.5 ± 44.0* | 4.3 ± 15.9* | 27.3 ± 42.0* | 1.8 ± 9.9* | 10.5 ± 29.7* | 20.1 |
|  | Control | 23.9 ± 201.3 | 7.5 ± 34.2 | 1.3 ± 5.6 | 18.2 ± 32.1 | 1.4 ± 19.3 | 5.8 ± 18.0 | NA |
| Y3-5 | Case | 86.6 ± 234.2* | 21.2 ± 40.7* | 4.0 ± 10.2* | 28.8 ± 42.5* | 1.4 ± 6.9* | 10.6 ± 26.8* | 19.4 |
|  | Control | 27.0 ± 99.9 | 7.5 ± 18.2 | 1.7 ± 5.5 | 19.1 ± 29.7 | 0.7 ± 2.0 | 6.3 ± 21.9 | NA |
| Y6-10 | Case | 64.5 ± 199.0* | 17.0 ± 40.1* | 4.1 ± 9.3* | 28.1 ± 38.9* | 1.2 ± 6.5* | 11.0 ± 25.6* | 18.0 |
|  | Control | 28.5 ± 87.5 | 8.6 ± 19.8 | 2.0 ± 5.3 | 18.5 ± 30.9 | 0.7 ± 2.7 | 6.5 ± 21.0 | NA |

Mann-Whitney U test was used to test for difference between case and controls. Statistical significance was based on group medians while means are presented as they are required for costing analyses. NA: not applicable; SD: standard deviation; SEK: Swedish krona; Y: year. ^§^: please refer to Table S6 for standard deviations of ostomy products cost (cost of ostomy solutions and supporting products); *: *p* < 0.0001.

Table S4 The 20 most common reasons for: A. 30-day readmission after colostomy creation (after a minimum of one day following discharge), B. Hospital admission during the first year after colostomy creation, and C. Hospital admission during the entire study period (primary and secondary diagnosis).

| A. 30-day readmission (total number of visits 4977 and 3873 persons) | | Number of persons with diagnosis | Number of visits with diagnosis | Share of total number of visits |
| --- | --- | --- | --- | --- |
| 1 | C78 Secondary malignant neoplasm of respiratory and digestive organs | 659 | 1061 | 21.3% |
| 2 | C20 Malignant neoplasm of rectum | 848 | 1043 | 21.0% |
| 3 | I10 Essential (primary) hypertension | 853 | 1023 | 20.6% |
| 4 | Z93 Artificial opening status | 771 | 961 | 19.3% |
| 5 | C18 Malignant neoplasm of colon | 618 | 781 | 15.7% |
| 6 | T81 Complications of procedures, not elsewhere classified | 580 | 704 | 14.1% |
| 7 | Z51 Encounter for other aftercare and medical care | 487 | 553 | 11.1% |
| 8 | Z85 Personal history of malignant neoplasm | 387 | 486 | 9.8% |
| 9 | E11 Type 2 diabetes mellitus | 378 | 480 | 9.6% |
| 10 | I48 Atrial fibrillation and flutter | 376 | 462 | 9.3% |
| 11 | C79 Secondary malignant neoplasm of other and unspecified sites | 250 | 358 | 7.2% |
| 12 | K56 Paralytic ileus and intestinal obstruction without hernia | 270 | 312 | 6.3% |
| 13 | Z92 Personal history of medical treatment | 236 | 285 | 5.7% |
| 14 | I25 Chronic ischemic heart disease | 209 | 281 | 5.6% |
| 15 | I50 Heart failure | 212 | 261 | 5.2% |
| 16 | C56 Malignant neoplasm of ovary | 188 | 259 | 5.2% |
| 17 | N39 Other disorders of urinary system | 246 | 257 | 5.2% |
| 18 | J44 Other chronic obstructive pulmonary disease | 190 | 248 | 5.0% |
| 19 | C77 Secondary and unspecified malignant neoplasm of lymph nodes | 194 | 239 | 4.8% |
| 20 | C21 Malignant neoplasm of anus and anal canal | 163 | 220 | 4.4% |

| B. Hospital admission during the first year after colostomy creation (total number of visits 54706) | | Number of persons with diagnosis | Number of visits with diagnosis | Share of total number of visits |
| --- | --- | --- | --- | --- |
| 1 | C78 Secondary malignant neoplasm of respiratory and digestive organs | 4096 | 12785 | 23.4% |
| 2 | C20 Malignant neoplasm of rectum | 5521 | 12194 | 22.3% |
| 3 | I10 Essential (primary) hypertension | 6375 | 11952 | 21.8% |
| 4 | C18 Malignant neoplasm of colon | 4022 | 8605 | 15.7% |
| 5 | Z93 Artificial opening status | 4144 | 7898 | 14.4% |
| 6 | Z85 Personal history of malignant neoplasm | 2915 | 5934 | 10.8% |
| 7 | K56 Paralytic ileus and intestinal obstruction without hernia | 4276 | 5781 | 10.6% |
| 8 | T81 Complications of procedures, not elsewhere classified | 3734 | 5559 | 10.2% |
| 9 | I48 Atrial fibrillation and flutter | 2424 | 5328 | 9.7% |
| 10 | E11 Type 2 diabetes mellitus | 2291 | 5302 | 9.7% |
| 11 | K57 Diverticular disease of intestine | 3697 | 5138 | 9.4% |
| 12 | Z51 Encounter for other aftercare and medical care | 2045 | 3698 | 6.8% |
| 13 | Z92 Personal history of medical treatment | 2068 | 3636 | 6.6% |
| 14 | C79 Secondary malignant neoplasm of other unspecified sites | 1440 | 3542 | 6.5% |
| 15 | I25 Chronic ischemic heart disease | 1495 | 3265 | 6.0% |
| 16 | I50 Heart failure | 1470 | 3030 | 5.5% |
| 17 | J44 Other chronic obstructive pulmonary disease | 1135 | 2661 | 4.9% |
| 18 | C77 Secondary and unspecified malignant neoplasm of lymph nodes | 1389 | 2454 | 4.5% |
| 19 | N39 Other disorders of urinary system | 1839 | 2364 | 4.3% |
| 20 | C56 Malignant neoplasm of ovary | 673 | 2262 | 4.1% |

| C. Hospital admission during the entire study period after colostomy creation (total number of visits 93247) | | Number of persons with diagnosis | Number of visits with diagnosis | Share of total number of visits |
| --- | --- | --- | --- | --- |
| 1 | C78 Secondary malignant neoplasm of respiratory and digestive organs | 5095 | 20863 | 22.4% |
| 2 | I10 Essential (primary) hypertension | 7507 | 20363 | 21.8% |
| 3 | Z93 Artificial opening status | 6621 | 18362 | 19.7% |
| 4 | Z85 Personal history of malignant neoplasm | 4864 | 15515 | 16.6% |
| 5 | C20 Malignant neoplasm of rectum | 5630 | 15122 | 16.2% |
| 6 | C18 Malignant neoplasm of colon | 4232 | 10826 | 11.6% |
| 7 | I48 Atrial fibrillation and flutter | 3122 | 10606 | 11.4% |
| 8 | E11 Type 2 diabetes mellitus | 2746 | 10268 | 11.0% |
| 9 | K56 Paralytic ileus and intestinal obstruction without hernia | 5299 | 8775 | 9.4% |
| 10 | Z92 Personal history of medical treatment | 2826 | 7022 | 7.5% |
| 11 | I50 Heart failure | 2217 | 6844 | 7.3% |
| 12 | T81 Complications of procedures, not elsewhere classified | 4149 | 6623 | 7.1% |
| 13 | I25 Chronic ischemic heart disease | 1950 | 6591 | 7.1% |
| 14 | C79 Secondary malignant neoplasm of other and unspecified sites | 2096 | 5979 | 6.4% |
| 15 | Z51 Encounter for other aftercare and medical care | 2868 | 5708 | 6.1% |
| 16 | J44 Other chronic obstructive pulmonary disease | 1426 | 5608 | 6.0% |
| 17 | K57 Diverticular disease of intestine | 3765 | 5527 | 5.9% |
| 18 | Z95 Presence of cardiac and vascular implants and grafts | 1417 | 4812 | 5.2% |
| 19 | N39 Other disorders of urinary system | 3008 | 4730 | 5.1% |
| 20 | Z86 Personal history of certain other diseases | 1656 | 4087 | 4.4% |

Table S5 The 20 most common reasons for: A. 30-day readmission after ileostomy creation (after a minimum of one day following discharge), B. Hospital admission during the first year after ileostomy creation, and C. Hospital admission during the entire study period (primary and secondary diagnosis).

| A. 30-day readmission after ileostomy creation (total number of visits 5739 and 4256 persons) | | Number of persons with diagnosis | Number of visits with diagnosis | Share of total number of visits |
| --- | --- | --- | --- | --- |
| 1 | Z93 Artificial opening status | 1048 | 1307 | 22.8% |
| 2 | I10 Essential (primary) hypertension | 934 | 1154 | 20.1% |
| 3 | C20 Malign neoplasm of rectum | 902 | 1113 | 19.4% |
| 4 | C18 Malign neoplasm of colon | 756 | 1017 | 17.7% |
| 5 | C78 Secondary malignant neoplasm of respiratory and digestive organs | 578 | 940 | 16.4% |
| 6 | T81 Complications of procedures, not elsewhere classified | 687 | 823 | 14.3% |
| 7 | E86 Dehydration | 644 | 746 | 13.0% |
| 8 | Z85 Personal history of malignant neoplasm | 554 | 733 | 12.8% |
| 9 | E87 Other disorders of fluid, electrolyte, and acid-base balance | 496 | 724 | 12.6% |
| 10 | E11 Type 2 diabetes mellitus | 458 | 614 | 10.7% |
| 11 | K56 Paralytic ileus and intestinal obstruction without hernia | 415 | 486 | 8.5% |
| 12 | N17 Acute kidney failure | 339 | 439 | 7.6% |
| 13 | I48 Atrial fibrillation and flutter | 316 | 416 | 7.2% |
| 14 | K51 Ulcerative colitis | 323 | 409 | 7.1% |
| 15 | K52 Other noninfective gastroenteritis and colitis | 293 | 348 | 6.1% |
| 16 | K91 Postprocedural disorders of digestive system, not elsewhere classified | 255 | 304 | 5.3% |
| 17 | K50 Crohn´s disease | 204 | 287 | 5.0% |
| 18 | Z92 Personal history of medical treatment | 206 | 267 | 4.7% |
| 19 | R10 Abdominal and pelvic pain | 246 | 266 | 4.6% |
| 20 | Z51 Encounter for other aftercare and medical care | 217 | 251 | 4.4% |

| B. Hospital admission during the first year after ileostomy creation (total number of visits 43847) | | Number of persons with diagnosis | Number of visits with diagnosis | Share of total number of visits |
| --- | --- | --- | --- | --- |
| 1 | C20 Malignant neoplasm of rectum | 5580 | 9539 | 21.8% |
| 2 | C78 Secondary malignant neoplasm of respiratory and digestive organs | 2729 | 8787 | 20.0% |
| 3 | C18 Malignant neoplasm of colon | 3575 | 8506 | 19.4% |
| 4 | I10 Essential (primary) hypertension | 4326 | 8117 | 18.5% |
| 5 | Z93 Artificial opening status | 3684 | 6915 | 15.8% |
| 6 | T81 Complications of procedures, not elsewhere classified | 3527 | 5278 | 12.0% |
| 7 | K56 Paralytic ileus and intestinal obstruction without hernia | 3759 | 5231 | 11.9% |
| 8 | Z85 Personal history of malignant neoplasm | 2445 | 5059 | 11.5% |
| 9 | K51 Ulcerative colitis | 2376 | 4318 | 9.8% |
| 10 | E11 Type 2 diabetes mellitus | 1586 | 3745 | 8.5% |
| 11 | I48 Atrial fibrillation and flutter | 1398 | 3153 | 7.2% |
| 12 | E87 Other disorders of fluid, electrolyte, and acid-base balance | 1567 | 2644 | 6.0% |
| 13 | Z92 Personal history of medical treatment | 1465 | 2449 | 5.6% |
| 14 | K50 Crohn´s disease | 981 | 2145 | 4.9% |
| 15 | E86 Dehydration | 1458 | 2068 | 4.7% |
| 16 | C77 Secondary and unspecified malignant neoplasm of lymph nodes | 1095 | 1934 | 4.4% |
| 17 | Z51 Encounter for other aftercare and medical care | 1110 | 1932 | 4.4% |
| 18 | I25 Chronic ischemic heart disease | 895 | 1876 | 4.3% |
| 19 | C56 Malignant neoplasm of ovary | 510 | 1817 | 4.1% |
| 20 | N17 Acute kidney failure | 1055 | 1655 | 3.8% |

| C. Hospital admission during the entire study period after ileostomy creation (total number of visits 62684 | | Number of persons with diagnosis | Number of visits with diagnosis | Share of total number of visits |
| --- | --- | --- | --- | --- |
| 1 | Z93 Artificial opening status | 4918 | 12705 | 20.3% |
| 2 | C78 Secondary malignant neoplasm of respiratory and digestive organs | 3071 | 12385 | 19.8% |
| 3 | I10 Essential (primary) hypertension | 4657 | 10857 | 17.3% |
| 4 | C20 Malignant neoplasm of rectum | 5613 | 10391 | 16.6% |
| 5 | C18 Malignant neoplasm of colon | 3626 | 9680 | 15.4% |
| 6 | Z85 Personal history of malignant neoplasm | 3148 | 9053 | 14.4% |
| 7 | K56 Paralytic ileus and intestinal obstruction without hernia | 4385 | 7095 | 11.3% |
| 8 | K51 Ulcerative colitis | 2446 | 6753 | 10.8% |
| 9 | T81 Complications of procedures, not elsewhere classified | 3834 | 6010 | 9.6% |
| 10 | E11 Type 2 diabetes mellitus | 1757 | 5876 | 9.4% |
| 11 | I48 Atrial fibrillation and flutter | 1592 | 4830 | 7.7% |
| 12 | K50 Crohn´s disease | 1076 | 3822 | 6.1% |
| 13 | Z92 Personal history of medical treatment | 1759 | 3668 | 5.9% |
| 14 | E87 Other disorders of fluid, electrolyte and acid-base balance | 1866 | 3471 | 5.5% |
| 15 | I25 Chronic ischemic heart disease | 1052 | 3147 | 5.0% |
| 16 | E86 Dehydration | 1843 | 2840 | 4.5% |
| 17 | Z51 Encounter for other aftercare and medical care | 1433 | 2740 | 4.4% |
| 18 | J44 Other chronic obstructive pulmonary disease | 730 | 2679 | 4.3% |
| 19 | C77 Secondary and unspecified malignant neoplasm of lymph nodes | 1249 | 2468 | 3.9% |
| 20 | Z95 Presence of cardiac and vascular implants and grafts | 803 | 2390 | 3.8% |

Table S6 The 20 most common reasons for: A. 30-day readmission after urostomy creation (after a minimum of one day following discharge), B. Hospital admission during the first year after urostomy creation, and C. Hospital admission during the entire study period (primary and secondary diagnosis).

| A. 30-day readmission after urostomy creation (total number of visits 1419 and 1095 persons) | | Number of persons with diagnosis | Number of visits with diagnosis | Share of total number of visits |
| --- | --- | --- | --- | --- |
| 1 | C67 Malignant neoplasm of bladder | 642 | 799 | 56.3% |
| 2 | T81 Complications of procedures, not elsewhere classified | 264 | 312 | 22.0% |
| 3 | I10 Essential (primary) hypertension | 267 | 308 | 21.7% |
| 4 | Z93 Artificial opening status | 233 | 280 | 19.7% |
| 5 | Z85 Personal history of malignant neoplasm | 192 | 266 | 18.7% |
| 6 | N39 Other disorders of urinary system | 213 | 245 | 17.3% |
| 7 | E11 Type 2 diabetes mellitus | 132 | 167 | 11.8% |
| 8 | A41 Other sepsis | 90 | 109 | 7.7% |
| 9 | Z92 Personal history of medical treatment | 89 | 99 | 7.0% |
| 10 | I48 Atrial fibrillation and flutter | 79 | 97 | 6.8% |
| 11 | E87 Other disorders of fluid, electrolyte, and acid-base balance | 67 | 94 | 6.6% |
| 12 | B96 Other specified bacterial agents as the cause of diseases classified elsewhere | 73 | 90 | 6.3% |
| 13 | K56 Paralytic ileus and intestinal obstruction without hernia | 69 | 85 | 6.0% |
| 14 | I25 Chronic ischemic heart disease | 68 | 80 | 5.6% |
| 15 | C61 Malignant neoplasm of prostate | 68 | 77 | 5.4% |
| 16 | N10 Acute tubulo-interstitial nephritis | 65 | 69 | 4.9% |
| 17 | N13 Obstructive and reflux uropathy | 60 | 68 | 4.8% |
| 18 | K59 Other functional intestinal disorders | 55 | 63 | 4.4% |
| 19 | J44 Other chronic obstructive pulmonary disease | 49 | 59 | 4.2% |
| 20 | Z86 Personal history of certain other diseases | 48 | 58 | 4.1% |

| B. Hospital admission during the first year after urostomy creation (total number of visits 13906) | | Number of persons with diagnosis | Number of visits with diagnosis | Share of total number of visits |
| --- | --- | --- | --- | --- |
| 1 | C67 Malignant neoplasm of bladder | 4137 | 9118 | 65.6% |
| 2 | I10 Essential (primary) hypertension | 1802 | 3415 | 24.6% |
| 3 | Z85 Personal history of malignant neoplasm | 1087 | 2374 | 17.1% |
| 4 | Z93 Artificial opening status | 1009 | 2158 | 15.5% |
| 5 | T81 Complications of procedures, not elsewhere classified | 1046 | 1541 | 11.1% |
| 6 | E11 Type 2 diabetes mellitus | 596 | 1429 | 10.3% |
| 7 | N39 Other disorders of urinary system | 884 | 1286 | 9.2% |
| 8 | Z92 Personal history of medical treatment | 638 | 1093 | 7.9% |
| 9 | C61 Malignant neoplasm of prostate | 622 | 1059 | 7.6% |
| 10 | C77 Secondary and unspecified malignant neoplasm of lymph nodes | 487 | 1051 | 7.6% |
| 11 | C79 Secondary malignant neoplasm of other and unspecified sites | 456 | 1042 | 7.5% |
| 12 | I48 Atrial fibrillation and flutter | 457 | 966 | 6.9% |
| 13 | C78 Secondary malignant neoplasm of respiratory and digestive organs | 403 | 946 | 6.8% |
| 14 | Z51 Encounter for other aftercare and medical care | 414 | 942 | 6.8% |
| 15 | I25 Chronic ischemic heart disease | 383 | 827 | 5.9% |
| 16 | N13 Obstructive and reflux uropathy | 449 | 765 | 5.5% |
| 17 | K56 Paralytic ileus and intestinal obstruction without hernia | 502 | 723 | 5.2% |
| 18 | J44 Other chronic obstructive pulmonary disease | 295 | 666 | 4.8% |
| 19 | Z95 Presence of cardiac and vascular implants and grafts | 269 | 556 | 4.0% |
| 20 | Z86 Personal history of certain other diseases | 292 | 538 | 3.9% |

| C. Hospital admission during the entire study period after urostomy creation (total number of visits 26846) | | Number of persons with diagnosis | Number of visits with diagnosis | Share of total number of visits |
| --- | --- | --- | --- | --- |
| 1 | C67 Malignant neoplasm of bladder | 4158 | 11723 | 43.7% |
| 2 | Z85 Personal history of malignant neoplasm | 2032 | 7289 | 27.2% |
| 3 | I10 Essential (primary) hypertension | 2180 | 6241 | 23.2% |
| 4 | Z93 Artificial opening status | 1807 | 5734 | 21.4% |
| 5 | E11 Type 2 diabetes mellitus | 783 | 3093 | 11.5% |
| 6 | N39 Other disorders of urinary system | 1510 | 2810 | 10.5% |
| 7 | C79 Secondary malignant neoplasm of other and unspecified | 842 | 2499 | 9.3% |
| 8 | C78 Secondary malignant neoplasm of respiratory and digestive organs | 801 | 2406 | 9.0% |
| 9 | I48 Atrial fibrillation and flutter | 683 | 2387 | 8.9% |
| 10 | Z92 Personal history of medical treatment | 935 | 2296 | 8.6% |
| 11 | I25 Chronic ischemic heart disease | 549 | 2077 | 7.7% |
| 12 | C77 Secondary and unspecified malignant neoplasm of lymph nodes | 723 | 1981 | 7.4% |
| 13 | T81 Complications of procedures, not elsewhere classified | 1212 | 1907 | 7.1% |
| 14 | Z51 Encounter for other aftercare and medical care | 732 | 1676 | 6.2% |
| 15 | C61 Malignant neoplasm of prostate | 717 | 1606 | 6.0% |
| 16 | B96 Other specified bacterial agents as the cause of diseases classified elsewhere | 815 | 1525 | 5.7% |
| 17 | J44 Other chronic obstructive pulmonary disease | 405 | 1506 | 5.6% |
| 18 | N13 Obstructive and reflux uropathy | 691 | 1494 | 5.6% |
| 19 | N18 Chronic kidney disease | 413 | 1453 | 5.4% |
| 20 | K56 Paralytic ileus and intestinal obstruction without hernia | 797 | 1446 | 5.4% |

Table S7 Mean annual cost per person of ostomy solutions and supporting products presented in 1000 SEK.

|  | Ostomy solutions | Ostomy supporting products |
| --- | --- | --- |
| Colostomy, mean ± SD | | |
| Y1 | 23.1 ± 13.2 | 5.0 ± 6.0 |
| Y2 | 19.7 ± 15.0 | 3.7 ± 6.0 |
| Y3-5 | 19.0 ± 14.1 | 3.6 ± 5.9 |
| Y6-10 | 19.3 ± 17.7 | 3.3 ± 5.6 |
| Ileostomy, mean ± SD | | |
| Y1 | 18.6 ± 11.3 | 7.4 ± 7.9 |
| Y2 | 14.5 ± 12.7 | 5.6 ± 8.2 |
| Y3-5 | 14.5 ± 11.4 | 5.4 ± 7.5 |
| Y6-10 | 14.7 ± 11.6 | 5.4 ± 11.2 |
| Urostomy, mean ± SD | | |
| Y1 | 20.6 ± 10.3 | 5.8 ± 5.3 |
| Y2 | 16.1 ± 10.5 | 4.0 ± 4.6 |
| Y3-5 | 15.3 ± 10.8 | 4.1 ± 5.7 |
| Y6-10 | 14.1 ± 9.6 | 3.9 ± 5.3 |

SD: standard deviation; SEK: Swedish krona; Y: year.

Table S8 Mean annual indirect costs per person after ostomy creation presented in 1000 SEK.

|  |  | Early retirement | Sickness absence |
| --- | --- | --- | --- |
| Colostomy, mean ± SD | | | |
| Y1 | Case | 9.5 ± 34.4* | 31.0 ± 73.3* |
|  | Control | 4.7 ± 24.5 | 2.3 ± 17.2 |
| Y2 | Case | 10.4 ± 36.5* | 18.9 ± 60.2* |
|  | Control | 4.4 ± 24.1 | 2.1 ± 16.9 |
| Y3-5 | Case | 10.5 ± 36.1* | 12.1 ± 47.1* |
|  | Control | 3.5 ± 20.5 | 2.1 ± 15.8 |
| Y6-10 | Case | 10.4 ± 34.9* | 5.3 ± 28.8^#^ |
|  | Control | 3.2 ± 19.3 | 2.0 ± 14.0 |
| Ileostomy, mean ± SD | | | |
| Y1 | Case | 8.7 ± 32.8* | 50.7 ± 85.8* |
|  | Control | 5.8 ± 27.0 | 3.4 ± 20.9 |
| Y2 | Case | 11.0 ± 37.0* | 33.0 ± 78.0* |
|  | Control | 5.6 ± 26.8 | 3.7 ± 24.0 |
| Y3-5 | Case | 12.1 ± 37.2* | 18.5 ± 52.3* |
|  | Control | 5.1 ± 24.8 | 3.4 ± 18.3 |
| Y6-10 | Case | 12.8 ± 37.3* | 9.0 ± 34.1* |
|  | Control | 4.5 ± 22.1 | 3.6 ± 17.8 |
| Urostomy, mean ± SD | | | |
| Y1 | Case | 8.7 ± 33.0* | 18.5 ± 52.8* |
|  | Control | 3.9 ± 22.0 | 1.8 ± 13.8 |
| Y2 | Case | 9.0 ± 33.8* | 9.8 ± 41.5* |
|  | Control | 3.6 ± 21.1 | 1.6 ± 14.1 |
| Y3-5 | Case | 9.0 ± 32.7* | 6.8 ± 33.2* |
|  | Control | 2.6 ± 17.3 | 1.5 ± 11.6 |
| Y6-10 | Case | 9.1 ± 32.3* | 1.9 ± 15.5 |
|  | Control | 1.5 ± 12.2 | 1.7 ± 13.1 |

Mann-Whitney U test was used to test for difference between case and controls. Statistical significance was based on group medians while means are presented as they are required for subsequent analyses. SD: standard deviation; SEK: Swedish krona; Y: year; *: *p* < 0.0001; ^#^: *p* = 0.0044.
